# The Microbiota–Gut–Brain Axis in Psychiatric Disorders

**DOI:** 10.3390/ijms231911245

**Published:** 2022-09-24

**Authors:** Aleksandra Góralczyk-Bińkowska, Dagmara Szmajda-Krygier, Elżbieta Kozłowska

**Affiliations:** 1 Department of Microbiology and Experimental Immunology, MOLecoLAB: Lodz Centre of Molecular Studies on Civilisation Diseases, Medical University of Lodz, Mazowiecka 5 Street, 92-215 Lodz, Poland; 2 Laboratory of Molecular Diagnostics and Pharmacogenomics, Department of Pharmaceutical Biochemistry and Molecular Diagnostics, Medical University of Lodz, Muszynskiego 1 Street, 90-151 Lodz, Poland

**Keywords:** host response, microbiota, major depressive disorder, schizophrenia, bipolar disorder, autism spectrum disorder, attention-deficit hyperactivity disorder

## Abstract

Modulating the gut microbiome and its influence on human health is the subject of intense research. The gut microbiota could be associated not only with gastroenterological diseases but also with psychiatric disorders. The importance of factors such as stress, mode of delivery, the role of probiotics, circadian clock system, diet, and occupational and environmental exposure in the relationship between the gut microbiota and brain function through bidirectional communication, described as “the microbiome–gut–brain axis”, is especially underlined. In this review, we discuss the link between the intestinal microbiome and the brain and host response involving different pathways between the intestinal microbiota and the nervous system (e.g., neurotransmitters, endocrine system, immunological mechanisms, or bacterial metabolites). We review the microbiota alterations and their results in the development of psychiatric disorders, including major depressive disorder (MDD), schizophrenia (SCZ), bipolar disorder (BD), autism spectrum disorder (ASD), and attention-deficit hyperactivity disorder (ADHD).

## 1. Introduction

Illness or mental disorder is a negative phenomenon for the patient, associated with disturbances in daily functioning, especially in fulfilling social roles. This is evident in affective disorders, in the course of psychotic disorders or autism spectrum disorder (ASD), in which emotional, cognitive, and communication disorders are associated with severe limitations in these people’s psychophysical and social competencies. Current predictions suggest that the problem of mental illnesses referring to people worldwide is significant and will only worsen [1]. Therefore, it is essential to understand the functioning of patients on a personal, social, and professional level and apply adequate psychiatric rehabilitation procedures. It seems even more important to look for answers to the question of the etiopathogenesis of these mysterious disorders. Such studies may contribute to changes in the field of prevention and promotion of mental health or the creation of more effective pharmacological agents and other medical interventions that can improve the functioning of people with mental disorders in all spheres of activity. In recent years, scientific attention has been paid to the relationship between neurological and psychiatric disorders and gut microbiota (GM) [2].

The microbiota is a group of microorganisms that colonize the human body, and the composition of this group is not accidental; in turn, the term microbiome covers the genomes of all microorganisms in a particular environment. This complex ecosystem is characterized by a complicated network of positive and negative interrelationships that significantly impact the host’s health [3]. Notably, the microbiota currently depends not only on the interdependencies occurring in this specific ecosystem but also on the cells of the macroorganism. Microorganisms inhabit various areas of the human body, ranging from the skin, through the mouth, the upper respiratory tract, the ear canal, and the vagina [4,5]. However, 90% of all microorganisms colonize the initial sections of the small and large intestines [6,7]. Many data indicate that the number of microorganisms that inhabit a macroorganism exceeds ten times the number of its cells.

Recently, the total weight of intestinal microbes was estimated at 1–2 kg [8,9]. According to Daniel’s studies [10], the wet weight of intestinal colon content reached approximately 200–250 g of colonic content, in which the bacteria representing the intestinal microbiome constituted about 100 g. It should also be noted that the composition of GM is very diverse. It is made up not only bacteria but also fungi (a good example is *Candida albicans*), viruses, and some protists [11]. An essential element of the intestinal microbiota are also microorganisms belonging to a separate kingdom of living organisms, Archaebacteria. These microorganisms occur in a wide variety, often in extreme environments, under strictly anaerobic conditions, obtaining energy from the transformation of simple inorganic or organic compounds (including in the process of methanogenesis, in which energy is obtained during the synthesis of methane from various substrates such as dioxide, carbon, and hydrogen) [12]. Due to variable pH conditions in individual sections of the gastrointestinal (GI) tract, oxygen conditions, access to nutrients, and also variable intestinal peristalsis, various sections of GI are colonized by specific microorganisms [3,13]. Any disturbance in the composition and quality of individual microbial communitiescan have serious health consequences. A characteristic and quite clear example that illustrates such disorders is SIBO (excessive bacteria in the small intestine) [14]. It might result from the impairment of gastric acid secretion, anatomical changes in the gastrointestinal tract as a result of various diseases (disorders of the ileocecal valve, intestinal diverticula, or intestinal tumors), abdominal cavity surgery (e.g., in the case of resection of a fragment of the large intestine in the course of a neoplastic disease), and also reduced intestinal motility in the course of various diseases or disorders (e.g., impairment of the function of the migrating myoelectric complex) [15,16].

The aim of this review is to discuss the microbiota–gut–brain axis relationships. We summarize the knowledge about pathways that are involved in this bidirectional communication. The importance of the difference factor that affects the gut microbiota is also underlined. In the end we present the results of GM correlation with common psychiatric disorders.

The methods of this review article were based on the use of the PubMed database, to search for all related published studies. The selection was based on the keywords, “gut”, “microbiome”, “microbiota”, “gut-brain axis”, “major depressive disorder”, “depression”, “MDD”, “bipolar disorder”, “BD”, “schizophrenia”, “SCZ”, “autism spectrum disorder”, “ASD”, “Attention-Deficit Hyperactivity Disorder”, and “ADHD”. The statistical data on the prevalence of psychiatric disorders described in this review were taken from the World Health Organization, Institute for Health Metrics and Evaluation, Our World in Data, and the Centers for Disease Control and Prevention.

## 2. Role of Intestinal Microbiota in the Host Organism

In the macroorganism, the intestinal microbiota fulfills many important functions. First, it maintains the proper functioning of the intestines, ensuring an appropriate pH, proper intestinal peristalsis, and a regular rhythm of bowel movement. Microorganisms colonizing the intestines not only participate in the digestion of food by secreting digestive enzymes or converting complex nutrients into simpler organic compounds and fat metabolism, but also participate in the absorption of digested food. In addition to the functions mentioned above, the intestinal microbiota is responsible for synthesizing vitamins, mainly those of group B [6,17]. Through anaerobic fermentation of indigestible carbohydrates (mostly dietary fiber), intestinal microorganisms produce short-chain fatty acids (SCFAs), which are the primary source of energy for an epithelial cell of the colon (colonocytes) [18]. Butyric acid plays the most important role in the nourishment of these cells while at the same time being an important factor in stimulating their growth and differentiation [19]. Another important role of the intestinal microbiota is the neutralization of toxins and carcinogenic compounds [20].

Furthermore, intestinal microorganisms create the intestinal barrier, thus protecting the macroorganism against the penetration of pathogenic factors [21]. It should be pointed out that the intestinal microbiota significantly affects the activity and functioning of the immune system—it has immunomodulatory functions, regulates the levels of cytokines through interaction with the lymphatic tissue of the digestive tract, and it is considered the largest lymphatic organ in the human body [22]. Taking the above into account, there is no doubt that all disturbances in the amount and composition of the intestinal microbiota (intestinal dysbiosis) lead to numerous abnormalities such as disruption of intestinal peristalsis, disturbances in digestion and absorption, disorders in vitamin production or metabolism, and difficulties in digesting fats, but also the destruction of the intestinal barrier and excessive stimulation of the immune system [7,23]. Meta-analysis of GM composition in psychiatric disorders performed by McGuinness et al. [24] revealed that there were no strong differences in the number or distribution (α-diversity) of intestinal bacteria. Still, in the case of major depressive disorder (MDD), schizophrenia (SCZ), and bipolar disorder (BD), after comparing them to controls, there were compositional differences (β-diversity). Based on work to date, some research models have been used in studies on the impact of intestinal microbiota on brain development and brain function. Such studies include experiments using germ-free mice (GF) that were raised under completely sterile conditions, without any intestinal microbiota [25,26], research with the use of probiotic and antibiotic therapy [27], studies on the use of fecal microbiota transplantation (FMT) [28], and the use of infectious research [29].

## 3. Factors Affecting Gut Microbiota

The connection between GM and the central nervous system (CNS) through bidirectional communication begins during intrauterine life and is affected by many intrinsic and extrinsic factors, such as vaginal/caesarean birth, lifestyle habits, living arrangements (urban or rural), dietary and medicament intake, and the host’s circadian clock [30,31,32,33].

### 3.1. Mode of Delivery

For a long time, it has been assumed that according to the “sterile womb dogma”, the human fetus is sterile until delivery and microbes start to colonize the human GI after birth, but some studies indicate the beginning of the infant microbiota colonization in utero [34,35,36]. This colonization of the infant by *Escherichia coli*, *Enterococcus faecium*, and *Staphylococcus epidermidis* could be associated with its translocation from the mother’s gut through the bloodstream and placenta [37]. According to Collado et al. [38], the microbiota observed in the placenta and the amniotic fluid harbor were characterized by low richness and diversity with the predominance of Proteobacteria.

The critical factor affecting newborns’ colonization of the GI tract is the mode of delivery [39,40]. During recent decades, despite the lack of medical recommendations, the number of cesarean sections (CS) worldwide has increased. In some countries, more than 50% of births occur in this way [41]. Studies have shown that the composition of the intestinal microbiota in vaginally delivered (VD) infants appears to be similar to that of the maternal vaginal microbiota: *Lactobacillus* dominates, followed by *Senathia* spp. and *Prevotella*, most of which are anaerobic bacteria. CS leads to an imbalance of the infant gut microbiota and decreased diversity. Due to the lack of opportunity to encounter the maternal vaginal microbiota, the hospital environment and the mother’s skin constitute their first contact. As a result, pathogens (*Enterococcus*, *Enterobacter*, and *Klebsiella*) from the hospital environment have been found in their intestines [42]. The intestinal microbiota of the infant delivered by CS contains a lower abundance of *Bifidobacteria, Bacteroides, Staphylococcus, Corynebacterium*, and a *Propionibacterium* spp. A higher quantity of *Lactobacillus*, *Prevotella*, *Sneathia* spp., and *Clostridium difficile* compared to VD children was found [39]. It should be mentioned that a high abundance of *C. difficile* could cause dysbiosis and an increased risk of developing obesity [43]. Therefore, GM colonization appears to be important for the health and development of the infant due to its proper development of metabolism, immune system function, and the brain in the following stages of life. Interestingly, the method of feeding can also influence the development of specific bacterial strains in the infant’s GM, for example, *Bifidobacterium longum*, by using oligosaccharides in mother’s milk, competes with *E. coli* and *Clostridium perfringens* [44]. Furthermore, primary studies revealed that *Lactobacillus acidophilus* LB might reduce necrotizing enterocolitis in preterm infants [45]. Therefore, more research on probiotic, prebiotic, and synbiotic supplementation in infant diet is essential to ensure optimal colonization of the infant’s intestine by microbes [46]. There is also an idea indicating an association between stress, the vaginal microbiota of a pregnant woman, and the development of the nervous system in her infant [47]. Exposure of pregnant women to stress could influence the development of the nervous system of their offspring by changing the composition of the vaginal microbiota. This provides the disturbances in the development of the intestinal microbiome in a newborn, thus affecting not only the functioning and development of the digestive system, but also the nervous and immune systems [48,49].

### 3.2. Probiotics

Currently, the growing number of data indicate probiotics as the form of treatment of mental disorders/neurological and developmental disorders in which increased intestinal permeability has been demonstrated, i.e., depression, anxiety, autism, schizophrenia, or bipolar disorder [50]. The mode of action of probiotic microorganisms includes, among others, regulation of the immune system, production of SCAFs, or support of the gut barrier integrity [51]. Several studies revealed the diversity and specificity of probiotic strains in affecting the brain. According to the meta-analysis of Huang et al. [52], among people with depression, taking probiotics significantly alleviated its symptoms. In the study by Messaoudi et al. [53] involving a group of healthy human volunteers, it was shown that taking probiotics containing *Lactobacillus helveticus* R0052 and *B. longum* R0175 for 30 days alleviated symptoms of depression and anxiety, reflected in the reduced rates of the Hospital Anxiety and Depression Scale (HADS), compared to the control group of people taking placebo. Another analysis revealed that certain strains such as *B. longum*, *Bifidobacterium animals lactis*, *Streptococcus thermophilus*, *Lactobacillus bulgaricus*, *L. lactis*, and *L. helveticus* reduce stress levels and alleviate symptoms of depression [54]. Furthermore, the positive effects of taking probiotics were also observed in children with ASD. Critchfield et al. [55] showed that probiotic supplementation in children with ASD can decrease inflammation and alleviate behavioral disorders. There is limited research into the effects of probiotics in SCZ in the human model. Nevertheless, it should be noted that Severance et al. [56] indicated in patients with SCZ an association between gastrointestinal inflammation and food antigen-associated immune activation. Furthermore, in research conducted by Ghaderi et al. [57], it has been revealed that administration of vitamin D to patients with SCZ for 12 weeks in combination with probiotic strains such as *Lactobacillus reuteri*, *L. fermentum*, *L. acidophilus*, and *Bifidobacterium bifidum* (2 × 10^9^ each) resulted in beneficial effects on the general and total PANSS (Positive and Negative Syndrome Scale) score. Although the influence of prebiotics SCZ does not explain the molecular mechanism of action, and research in this area does not provide crystalline and unambiguous conclusions, there is a temptation to explore the topic of the influence of probiotic strains and prebiotics on the development and course of SCZ.

An interesting issue recently raised by scientists is the impact of probiotics on mood improvement during COVID-19. Probiotics, in addition to restoring intestinal balance, reduces risk of colonization of the intestine by opportunistic pathogens [58]. According to Rogers et al. [59], COVID-19 infection could result in posttraumatic stress disorder (PTSD). The dysbiosis of the gut microbiota affected by SARS-CoV-2 infection leads to improper transport of intestinal nutrients [60]. Gu et al. [61] showed the growth of the number of opportunistic pathogens such as *Streptococcus*, *Rothia*, *Veillonella*, and *Actinomyces* with a simultaneous decline in the number of helpful symbionts in patients with COVID-19 and H1N1 compared to healthy patients. Therefore, studies demonstrating probiotic supplementation and its impact on depressed mood caused by COVID-19 could prove to be highly useful. The survey by d’Ettorre et al. [62] revealed that the unique probiotic formulation (three doses of 2400 billion bacteria each per day) by patients with COVID-19 decreased the risk of severe course of the disease. This formulation consisted of *Streptococcus thermophilus* DSM 32345, *L. acidophilus* DSM 32241, *L. helveticus* DSM 32242, *L. paracasei* DSM 32243, *L. plantarum* DSM 32244, *L. brevis* DSM 27961, *B. lactis* DSM 32246, and *B. lactis* DSM 32247. However, the study of Slykerman and Li [63] covering nurses working during the 2020 pandemic year in New Zealand who took the probiotic *Lactobacillus rhamnosus* HN001 probiotic for 12 weeks have not revealed significant differences in recognized stress or signs of viral disease. It should also be noted that there are some limitations in probiotic supplementation; for example, they are not recommended for immunocompromised patients treated with corticosteroids [64]. Although there are no global approved ordinances on probiotic supplementation of probiotics in patients with COVID-19, probiotics could potentially find application in the prevention and complementary therapy of this disease.

### 3.3. Stress

Several reviews have also shown a relationship between stress and changes in the amount and composition of gut microbiota. In humans, stress has been shown to cause a radical decrease in the number of *Lactobacillus* spp. and *Bifidobacterium* spp. In turn, in mice exposed to stress, the number of *Lactobacillus* spp. was reduced [65], while the amount of bacteria from the genus *Clostridium* spp. increased. However, potentially due to the production and secretion of catecholamines, adrenaline, and norepinephrine, the number of pathogenic and non-pathogenic strains of *E. coli* increased.

Most psychological theories show a clear relationship between stress and the risk of developing a mental disorder or disease. For example, a significant association has been demonstrated between disturbances caused by stressful stimuli, especially those with long-term effects, and depression [66,67]. Various stressors, especially during childhood, increase the risk of developing mental illnesses, including affective and anxiety disorders. The dysfunction of the hypothalamic–pituitary–adrenal axis (HPA) is of great importance in the mechanism of this phenomenon. It is related to the increase in the release of corticotropin-releasing hormone (CRH), also known as corticotropin-releasing factor (CRF) or corticoliberin [31]. Arginine vasopressin (AVP), secreted together with CRH under stress, works synergistically. Thanks to discovering the directions and mechanisms of CRH activity, the knowledge about the endocrine system’s role in the etiopathogenesis of depression and how antidepressants work has increased [68]. CRH plays a vital role in the body’s response to stress stimuli, increasing the secretion of adrenocorticotropic hormone (ACTH) and cortisol [69]. Furthermore, it exhibits strong psychotropic effects, among which the predominant reactions are anxiety and depressive reactions and disturbances in regulating sleep and nutrition [70]. It is also essential that the nervous and endocrine systems constitute an inextricable, complementary, and mutually interacting structural and functional totality that performs regulatory functions in the body [71]. In depression, dysfunction of serotonergic (5-HT, 5-hydroxytryptamine) transmission, as well as increased activity and dysregulation of the limbic–hypothalamic–pituitary–adrenal axis (LHPA axis), are also observed [72]. These phenomena probably play an important role in their pathogenesis; therefore, this relationship seems to connect the previously separate hypotheses of depression: serotonin and glucocorticoid. They are the most popular biological pathogenetic concepts of affective disorders, indicating in the course of depression deficiency of following neurotransmitters such as catecholamines-noradrenaline (NA), dopamine (DA), and serotonin (5-HT). In recent years, it has also been shown that in affective disorder, besides disturbances in the field of neurotransmitters and neurohormones, there are specific structural changes in the central nervous system. These changes mainly affect the hippocampus and prefrontal cortex structures and consist of the reduction of the number of cells and the weakening of the processes of neurogenesis and neuronal plasticity [73]. Based on the review mentioned above, the results of genetic and molecular studies, as well as neurobiological data obtained through biochemical and neuroimaging studies, depression seems to be well known in terms of its etiopathogenesis and control and treatment methods. However, more and more people suffer from this severe disease, leading to a significant reduction in patient quality of life and many suicide attempts. Therefore, new concepts of affective disorders are possible that will allow for a new look at the pathogenesis and treatment of these diseases in the near future.

### 3.4. Circadian Clock System

An interesting aspect is also the connection between the gut microbiota and the host’s circadian clock. Circadian rhythm is an intrinsic rhythm that exists in most organisms and arranges different processes occurring in the whole body [74]. The circadian clock system comprises a central circadian clock, placed in the suprachiasmatic nucleus (SCN) of the hypothalamus, and peripheral circadian clocks, located in tissues such as the intestine, pancreas, heart, liver, skeletal muscles, and kidneys. GM has diurnal fluctuations that can be affected by phase shifting (jetlag), shift work, light, sleep, dietary nutrition, and stress [75,76,77]. As described in Section 3.3, stress has a tremendous effect on the human organism and causes dysregulation of the intestinal microbiota. Galley et al. [78] demonstrated that exposure to even 2 h of a social stressor, also known as social disruption, can affect microbial populations of the colonic mucosa. The changes included the decrease in relative and absolute abundance of the genus *Lactobacillus*, which has immunomodulatory functions in the colon, and probiotic action in treating inflammation. Some researchers indicated that psychiatric and metabolic disorders are associated with circadian rhythm [74]. Due to the interruption of the circadian clock system, brain signals entrain the peripheral clock of the gut and then cause gut microbiota dysbiosis, translocation of bacteria, and the development of inflammation with a higher risk for metabolic diseases [79]. According to Takaesu [80], factors such as shift work or evening chronotype (preference for activity in the evening) have been associated with increased frequency or intensification of BD and MDD [81].

There are also some reports demonstrating the regulation of human behavior in a circadian manner by a “clock genes” such as *Per, Cry, Bmal1*, and the *Clock* of host bacteria. Paulose et al. [76] revealed that the presence of melatonin, secreted in the GI tract, enlarged the quantity of swarming in *Enterobacter aerogenes* cultures. This commensal bacterium occurs in the human GI tract. Although the precise mechanism for this synchronization remains not fully understood, the data obtained illustrate that the circadian system of the host may regulate its microbiome through signals from bacterial clocks. The gut microbiota plays a crucial role in the liver’s clock reprogramming and circadian homeostasis [82]. Murakami et al. [83] demonstrated that, as a result of a high-fat diet (HFD), GM stimulates through peroxisome proliferator-activated receptor-γ (PPARγ) transcriptional reprogramming in the liver.

### 3.5. Occupational and Environmental Exposure

Another factor influencing GM is occupational exposure at work. Based on the earliest publications, researchers have focused mainly on workers in cotton textile and livestock farmers’ factories [84]. Due to the fact that individuals spend most of their life working, occupational exposure to chemical, physical, and biological hazards current in the workplace becomes an important factor in determining the microbiota [85]. According to the review of Mucci et al. [75], the most dangerous agents causing alterations in worker’s microbiome were exposure to biological agents (direct contact with animals and healthcare personnel), and chemical agents (metalworking fluids, dust, pesticides), following pressure of work, and changed nutritional habits as a result of long journeys and microclimate conditions. For example, after the sea voyage (30 days), the microbiome of sailors has changed, which led to an increase in the species *Streptococcus gordonii* and *Klebsiella pneumoniae* [86]. An interesting result was obtained according to the shift workers. In the samples from the night shift worker, apart from the rise of *Firmicutes* and *Actinobacteria* and decline of Bacteroidetes, a higher number of *Dorea longicatena* and *Dorea formicigenerans* was found. An abundance of *Faecalibacterium* was detected in day shift workers [79]. Such studies that indicate differences in microbiome composition and the presence of specific genera could serve as biomarkers in diagnosing and monitoring workers’ health [75].

In addition to occupational exposure, the environmental pollutants’ risk arose due to human activities, agriculture, and industry. Among xenobiotics (exogenous substances) are heavy metals, pesticides, herbicides, polycyclic aromatic hydrocarbons (PAHs), or polychlorinated biphenyls (PCBs) [87]. Heavy metals present in the environment can modify the composition of GM and thus affect human health [88]. The effect of xenobiotics on the microbiota and brain depends on the pollutants’ dose and exposure age. According to Voorhees et al. [89], early-life exposure to chlorpyrifos (CPF), an organophosphate pesticide, caused chronic microglial dysregulation and accelerated neurodegeneration in both males and females, thus increasing the risk of Alzheimer’s disease (AD). On the other hand, microorganisms have developed several mechanisms enabling biotransformation. Still, in some cases during such processes, end-or by-products show higher toxicity than the maternity compound [20]. PAHs, formed due to the incomplete burning of waste and fuels, are included in cigarette smoke and grilled meats. It has been shown that smoking leads to an increase in the proportion of the phylum of *Bacteroidetes* and a decrease in the phylum of *Firmicutes* and *Proteobacteria* in smokers compared to nonsmokers. However, after smoking cessation the GM needs time to alter, but, finally, it returns to the state of non-smokers. For that reason, quitting smoking remains the best solution in the treatment of diseases related to altered GM composition [90,91].

### 3.6. Diet

One of the key factors impacting the diversity and abundance of intestinal microbiota and simultaneously building immunologic reactions is diet [92]. Dietary nutrients, including vitamins, minerals, polyunsaturated fatty acids (PUFAs), and amino acids, are crucial for maintaining healthy brain structure and function. They participate in various metabolic pathways, cell signaling, glucose and lipid metabolism, and neurotransmitter synthesis processes [93]. SCFAs are products of microbiota metabolism based on nutrients delivered with food consumed by the host, especially dietary fiber (see more in Section 4.6). The study of Parletta et al. [93] revealed that the Mediterranean diet (MedDiet) supplemented with fish oil can reduce depressive symptoms compared to control conditions. Contrary to the statement that the Mediterranean diet is recommended for people suffering from depression, some research showed the opposite effect. In a review by Jain et al. [94], evidence of the impact of vegetarian and vegan diets on depression was discussed. Among the analyzed studies, 44% of the results revealed the connection between vegetarian and vegan diets with a higher risk of depression. In comparison, 28% of records showed the advantages of these diets on MDD, and 28% of work indicated no association between vegetarian and vegan diets and depression. Therefore, the evidence on the influence of vegetarian and vegan diets on depression remains inconclusive, and further studies are necessary.

Another example is the ketogenic diet, a high-fat, low-carbohydrate diet that modulates the body toward fat metabolism [95]. According to several clinical studies, a ketogenic diet showed a positive effect on patients with AD through elucidation of the level of high blood ketone, which improved cognition and memory [96]. Interestingly, there is also a hypothesis that in the fecal microbiota, the proportions of *Firmicutes* and *Bacteroidetes* differ between obese and lean humans [97]. However, Duncan et al. [98] has revealed no significant change in the Bacteroidets ratio in fecal samples of obese individuals during reduced-carbohydrate weight-loss diets. Furthermore, this analysis confirmed that response to diet (especially amount and type of carbohydrate) decreased the proportions of *Roseburia* and *Eubacterium rectale* group, responsible for butyrate production and probably colonic health maintenance. Therefore, the described changes of GM determined by diet affect the proper work of the colon and whole human metabolism, and further studies can better understand these dependencies.

## 4. The Pathways between Gut Microbiota and the Nervous System

**Figure 1:**
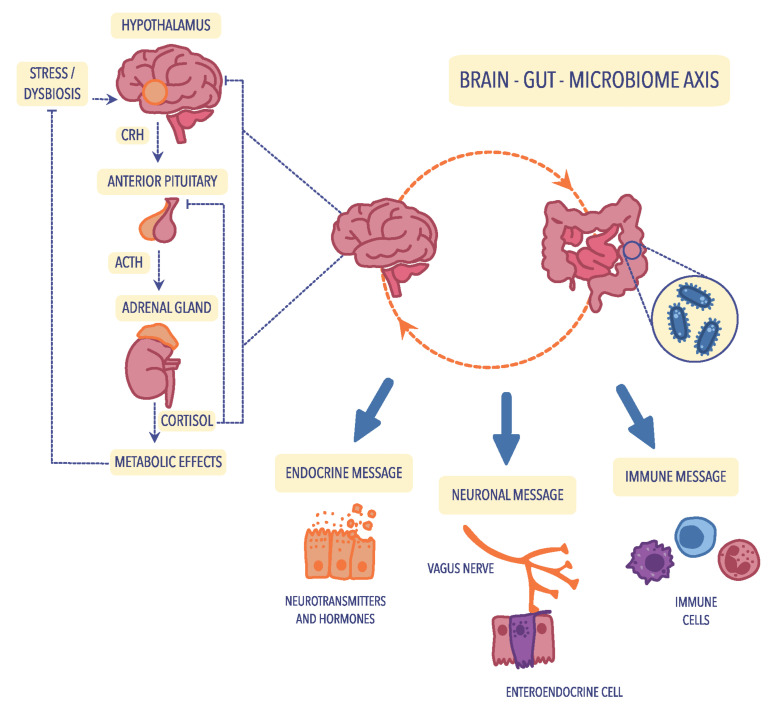
The bidirectional communication of the microbiota–gut–brain axis is mediated by the immune, neuroendocrine, and neuronal pathways. The activation of the hypothalamic–pituitary–adrenal (HPA) axis is associated with the occurrence of stress factors or dysbiosis, which increase the release of corticotropin-releasing hormone (CRH) from the hypothalamus, which subsequently stimulates the transport of adrenocorticotropic hormone (ACTH) from the anterior pituitary. Under the influence of ACTH, the adrenal gland begins to produce and secrete the stress hormone (cortisol), which is responsible for the modulation of intestinal immune and barrier functions. Figure was created using the Vectornator application.

As shown in Figure 1, the bidirectional connection between the gut and the brain is based on metabolic, endocrine, neural, and immunological pathways. It includes the vagal nerve, the HPA axis, the production of bacterial metabolites, immune mediators, and entero–endocrine signaling [50,99].

### 4.1. The Hypothalamic–Pituitary–Adrenal (HPA) Axis

The cerebral–intestinal axis is controlled at several levels, and the primary regulator is the nervous and endocrine (with the primary role of the HPA axis) and the immune pathway. Immune regulation of the HPA axis occurs mainly through the modification of cytokine secretion. In contrast, nervous regulation occurs primarily via the transmission of impulses in the autonomic nervous system, including the vagus nerve, afferent and centrifugal fibers, and the enteric nervous system (ENS). ENS, known as the “gut brain”, was first described in 1998 by Michael Gershon of Columbia University Medical Center [100]. The enteric nervous system is not only responsible for the direct regulation of muscles, mucosa, and vessels in the digestive tract but also for its activity. It comprises a large number of nerve fibers that form an impressive network of connections, and it is worth pointing out that over 30 different neurotransmitters are involved in the functioning of this system. There are about 40 neurons for each intestinal villi [101]. Contrary to the peripheral nervous system, the neuronal elements of the enteric nervous system are not surrounded by collagen and Schwann cells; instead, they are enveloped by glia that resemble CNS astrocytes. The ENS is formed by the Meissner plexus, located in the submucosa of the intestine, and the Auerbach plexus, located between the layer of circular and longitudinal muscles [102]. Due to this location, the ENS, through numerous transmitters and cytokines, remains in close contact with intestinal-associated lymphoid tissue (GALT) and the systemic humoral defense system of mucosa-associated lymphoid tissue (MALT). The neurotransmitters of the intestinal nervous system act, among others, on receptors in Peyer’s patches and lymphocytes. Most of GALT consists of lymphocytes of the entire immune system (70%), constituting the first line of defense and playing a particularly important role in the immune response to external antigens [103,104]. Likewise, microorganisms that inhabit the intestines, certain species of bacteria and fungi, transmit signals to both GALT and ENS through the synthesis and secretion of many different neurotransmitters [105]. Hormonal regulation of cerebral–intestinal communication occurs primarily through the HPA axis, also known as the stress axis, which primarily regulates the course of the stress response. Hypothalamic hormones-corticoliberin, together with vasopressin, start a hormonal cascade along the HPA axis, stimulating the anterior pituitary gland to produce and secrete the corticotropic hormone ACTH, which, along with the bloodstream, goes to the adrenal cortex and stimulates it to secrete glucocorticoids, mainly cortisol [69].

The cerebral–gut axis (which strongly connects the brain, intestines, and intestinal microbiota) is a two-way communication pathway, where brain–gut communication occurs through the autonomic nervous system (AUN), mainly the vagus nerve. Research has revealed that 90% of the impulses within the cerebral–intestinal axis are transmitted centripetally, i.e., from the intestines to the brain, and only 10% centrally. According to many observations, it has been proven that after vagotomy, the intestines continue to function correctly [101].

### 4.2. Neuroendocrine Pathways

Cortisol plays a key role in the endocrine mechanisms that regulate the gut–brain axis because it affects the cells of the immune system by modulating the secretion of cytokines that act on the HPA axis, but also significantly affects the functioning and differentiation of the intestinal microbiota [106]. On the other hand, special attention should be paid to the fact that intestinal bacteria produce numerous substances such as γ-aminobutyric acid (GABA) (*Lactobacillus* spp., *Bifidobacterium* spp.), acetylcholine (*Lactobacillus* spp.), serotonin (*Escherichia* spp., *Candida* spp., *Enterococcus* spp.), dopamine (*Bacillus* spp.), or noradrenaline (*Bacillus* spp., *Saccharomyces* spp.). These substances are involved not only in communication within the intestinal microflora but also in systemic and peripheral effects that affect brain functioning [107].

The intestinal microbiota also influences the level and metabolism of glutamic acid (glutamate), one of the main stimulants of the CNS. Glutamic acid is synthesized in glutamine-derived glial cells with the participation of glutaminase. The most important receptor of the glutamatergic system is the *N*-methyl-d-aspartate receptor (NMDA) [108]. Under physiological conditions, glutamic acid is the basis of learning and memory. Therefore, it has been widely applied to treating memory disorders and nervous exhaustion. However, an abundance of this acid causes an overactivation of glutamatergic receptors and consequently damages neurons. Both glutamine and glutamic acid are converted into GABA, the main inhibitory neurotransmitter, in other metabolic pathways. Several studies reported that the gut microbiota affects not only the level of GABA but also its metabolism [109].

### 4.3. Tryptophan Metabolism

**Figure 2:**
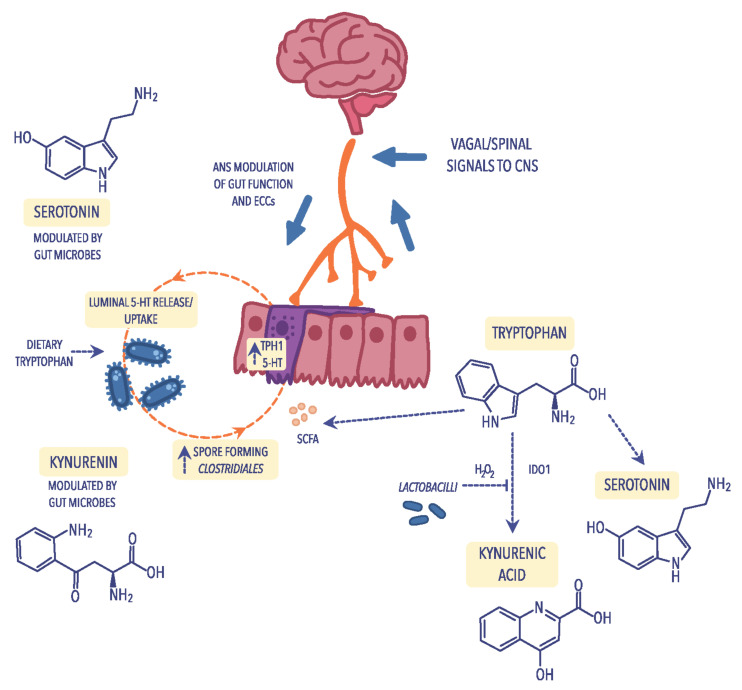
Metabolism of tryptophan through different pathways: the kynurenine pathway that leads to the production of kynurenine and its derivatives in reliance on indoleamine 2,3-dioxygenase (IDO); the serotonin pathway, including the enterochromaffin cells (ECCs) in which tryptophan is transformed into serotonin (5-HT) and its derivatives and is dependent on short-chain fatty acids (SCFAs) which are biosynthesized by spore-forming *Clostridiales*. ECCs can be activated by the autonomic nervous system (ANS) to secrete 5-HT into the gut lumen, where it can communicate with the intestinal microbiota. Figure was created using the Vectornator application.

A particular example of how the gut microbiota can influence the brain–gut axis is the synthesis of serotonin, commonly known as the “happiness hormone”. Serotonin deficiency in the CNS is one of the factors that cause depression, sadness, apathy, and anxiety and, according to modern concepts, is the leading cause of depressed mood. Therefore, drugs belonging to the class of selective serotonin reuptake inhibitors (SSRIs) have widespread use in treating mood disorders [110]. Serotonin is produced due to the transformation of tryptophan, one of the essential amino acids, of which about 2% of tryptophan ingested with food is converted to serotonin. In the body, serotonin is produced in the digestive tract, nervous system, and immune system (Figure 2) [111]. In the GI tract, even 95% of serotonin is produced by the mucosa’s enterochromatophilic cells (ECCs), microorganisms that are part of the intestinal microbiota, and neurons of nerve plexuses in the submucosa and muscular layers of the intestine [112]. Only 3% of serotonin is produced in thrombocytes and 2% in the pineal gland. The primary function of serotonin in the nervous system is neurotransmission, where serotonergic neurons play an important role in regulating pain, sleep, mood changes, and memory processes. In addition, serotonin is also an essential transmitter in the ENS [113]. Serotonin receptors in the GI tract are found not only in the neurons of the submucosa and muscle plexuses but also in enterocytes and smooth muscle cells. Through receptors, serotonin acts on the activity of the GI tract (both inhibiting and stimulating its function) [112]. Studies have revealed that altered serotonin metabolism is involved in the pathogenesis of certain GI diseases. In other words, in inflammatory bowel diseases, GI infections, and also in appendicitis, an increase in serum serotonin concentration has been observed [114]. Studies also demonstrated that the intestinal bacteria *Bifidobacterium infantis* affect the level and metabolism of tryptophan, thus increasing its level in the body [29].

### 4.4. Immunological Mechanisms

In recent years, the amount of evidence that points to an inflammatory basis of a psychiatric disorder has increased. There are indications that anti-inflammatory cytokines influence both neurohormonal and neurochemical functions in this process [115,116]. The primary factor leading to the systemic inflammatory response is increased intestinal permeability, also known as a leaky gut syndrome (LGS) [117]. Mechanisms involved in LGS include, first of all, disturbances in the intestinal microbiota within the GI tract, damage to enterocytes, weakening of tight connections between enterocytes, but also stress, which plays a particularly important role in the pathophysiology of depression [118]. As a result of the development of this syndrome, the translocation of Gram-negative bacteria containing lipopolysaccharide (LPS) causes overactivation of the immune system [96]. This stimulation causes an increase in the concentration of pro-inflammatory cytokines, the excess of which is destructive to host cells, including cells of the CNS. Since immune cells produce several cytokines, chemokines, and inflammatory mediators, leading to general inflammation of the organism, one of the hypotheses of the development of affective disorders is inflammation of the organism [119].

According to numerous observations, intestinal dysbiosis is the cause of the development of many intestinal diseases such as intestinal candidiasis, Crohn’s disease, ulcerative colitis, pseudomembranous enteritis, and inflammatory bowel disease, but also food allergies and intolerances, or colorectal cancer [120,121]. It should be noted that disturbances in the amount and composition of the intestinal microbiota promote the development of pathological conditions within the intestines and systemic diseases, allergies, obesity, metabolic syndrome, and autoimmune diseases. Therefore, the question can be asked whether the intestinal microbiota influences cognitive disorders and whether intestinal dysbiosis will affect the dysregulation of these processes? The answer is neither simple nor unambiguous. However, some premises, observations, and facts allow the formation of theories and hypotheses. The truth is that there is close communication between the brain and the intestines. It is known that the brain directly regulates the functioning of the intestines. However, special attention should be paid to the reverse direction of this communication. In particular, the processes in the intestines and the intestinal microbiota can affect the CNS’s functioning.

Pro-inflammatory cytokines also affect the concentration of serotonin by activating the kynurenine pathway in which tryptophan, the precursor of serotonin, is metabolized [122]. Increasing the concentration of pro-inflammatory cytokines activates indoleamine 2,3-dioxygenase (IDO), which results in a decrease in the concentration of tryptophan and serotonin (5-HT), simultaneously intensifying the symptoms of affective disorders and a rise in the level of tryptophan catabolites [29]. Under the influence of IDO, tryptophan is converted to kynurenine, quinolinic acid, and 3-hydroxy-kynurenine, which reduces serotonin production while increasing the concentration of toxic metabolites in the CNS [123,124]. Moreover, the weakened intestinal barrier allows the penetration of nutrients (food antigens). In addition, in contact with IgG antibodies, formed immune complexes are moved with the blood circulation, among others, for the choroid plexus of the CNS. Chronic accumulation of such complexes causes the initiation of inflammation and the progression of chronic ailments.

### 4.5. Bacterial Cell Wall Sugars

Some researchers emphasize that in healthy people, the dose of bacterial LPS may be associated with a more frequent occurrence of depression and anxiety and also contributes to an increase in norepinephrine and pro-inflammatory cytokines and cortisol in blood plasma and saliva, respectively [125,126]. Interestingly, some studies have shown some effects of LPS on emotional memory, suggesting that this ability depends on the dose [127]. Evidence in people with psychiatric disorders indicates decreased secretion of gastric acid that contributes to the development of small intestinal bacterial overgrowth (SIBO). SIBO is characterized by several symptoms that directly indicate changes in the composition and quality of the microbiota in individual sections of the intestine. The presence of bacteria in the small intestine, which live in the large intestine under physiological conditions, causes their physiological and metabolic processes to lead to numerous symptoms in the digestive system [14,128]. In addition, when decomposing proteins, bacteria such as *Clostridium, Proteus,* and *Enterobacteriaceae* produce ammonia, a potent neurotoxin. The high concentration of this substance in the blood leads to a deficiency of alpha-ketoglutaric acid, responsible for the removal of ammonia from the CNS and consequently causes a toxic effect in the enlargement of neurological symptoms [129]. It should be noted that some species of *Clostridium* can reduce the secretion of dopamine beta-hydroxylase, the enzyme responsible for converting dopamine to norepinephrine, resulting in a deficiency of norepinephrine and an excess of dopamine [130]. The imbalance of the mentioned compounds leads not only to the development of compulsive behaviors and obsessive states but also to their disturbed proportion, characteristic of SCZ or ASD [131].

### 4.6. Bacterial Metabolites

The products or metabolites of the gut, such as short-chain fatty acids, are carboxylic acids with aliphatic tails of 1 to 6 carbon atoms produced during anaerobic bacterial fermentation, mainly of dietary fiber [132]. More than 95% of SCFAs are butyric acid (butyrate), acetic acid (acetate), and propionic acid (propionate), although lactic acid was also found in smaller amounts [133]. Among SCFAs-producing bacteria there are *Clostridium* spp., *Eubacterium* spp., *Fusobacterium* spp., *Butyrivibrio* spp., *Megasphaera elsdenii*, *Mitsuokella multiacida*, *Rosburia intestinalis*, *Faecalibacterium prausnitzii*, and *Eubacterium halli* [26]. The primary source of energy for colonocytes is butyrate, which not only nourishes colonocytes but is also an essential factor that stimulates the growth and differentiation of these cells [19]. In addition, SCFAs play various roles in the human body. For example, they stimulate saprophytic microorganisms’ growth while inhibiting pathogenic bacteria such as *E. coli*, *Campylobacter* sp. Or *Salmonella* sp., which compete with saprophytic bacteria for the site of colonization [134]. They also accelerate healing and support intestinal epithelium, stimulate biosynthesis of mucus in the intestinal epithelium, and maintain the correct pH in the intestines, thus contributing to the protection of the GI tract against disturbances in the intestinal microbiota [135,136]. Furthermore, SCFAs support the maintenance of the proper intestinal barrier, e.g., by reducing the absorption of inulin, which stimulates the development of normal gut microbiota [137]. SCFAs also have an anti-inflammatory effect by inhibiting the activity of inflammatory mediators in the intestinal epithelium, reducing IL-8 secretion while blocking the anti-inflammatory cytokine cascade [138].

The absence of microorganisms that produce SCFAs is believed to cause multiple brain disorders. In studies in axenic mice, mice lacking bacterial microbiota exhibit severe disorders of microglial cell development [139]. In turn, in mice with intestinal dysbiosis, significant changes in the functioning of these cells were observed. After colonizing mice with the microbiota of a very diverse composition, a substantial improvement in the function of microglial cells was observed [140,141]. Interestingly, the administration of SCFAs to mice also improved the functioning of microglial cells. Furthermore, many changes in the neurotransmitter system and its receptors in various parts of the brain were observed in GF mice [142]. Additionally, a significant increase in serotonin levels was observed in the hippocampus and a reduced degree of receptor expression. It is noteworthy that GF mice showed in the cerebral cortex and amygdala a decreased expression of a brain-derived neurotrophic factor (BDNF) [8]. This neurotrophin has been shown to affect not only the differentiation and synaptic plasticity of neurons but also changes in different brain areas. BDNF is believed to be a factor that plays an important role in the development of mental diseases, especially the development of SCZ and MDD; however, its exact contribution to the development of these diseases is not fully known [109,143]. Żełobowska et al. [144] pointed to the reduced concentration of this active peptide as a common pathogenetic factor in cognitive impairment, depression, dementia, and type 2 diabetes. Based on samples from Alzheimer’s disease (AD) donors, decreased BDNF expression has been shown in the hippocampus, indicating its role in AD [145]. The lower level of BDNF protein in the blood serum compared to healthy people has been shown in patients with SCZ by Libman-Sokołowska et al. [146].

## 5. The Intestinal Microbiota in Neurological and Psychiatric Disorders

### 5.1. Depression (Major Depressive Disorder; MDD)

Depression is described as a common mental disorder state, characterized by a continuous feeling of sadness and apathy lasting at least two weeks, as a result of interactions covering social, psychological, and biological factors, for example, significant changes in life, family matters, chronic health problems, or addiction [147,148]. It is also a common cause of disability and a cause of suicide death. According to WHO, approximately 280 million people worldwide suffer from MDD and every year, and more than 700,000 people die from committing suicide [149]. The data described many factors that link this mental disorder with the components of the intestinal microbiota, which was confirmed by Naseribafrouei et al. [150]. They have shown that the level of the Alistipes genus associated with inflammation and *Oscillibacter*, which has valeric acid involved in is associated with depression, was elevated in patients with MDD.

Zhang et al. [151] showed, using a mouse model, that microbiota dysbiosis was associated with greater intestinal permeability and systemic inflammation. As a result of endogenous melatonin reduction (EMR), the composition of the mice microbiota changed and consisted of a decrease in the relative abundance of *Bacteroidetes*, an alteration of the *Firmicutes/Bacteroidetes* ratio, and growth of the relative abundance of *Lactobacillus*. The study also revealed improved gut permeability (leaky gut) and systemic inflammation in EMR mice.

The determination of SCFAs content could be helpful in the analysis of the microbiota composition by patients with MDD. The study concerning the SCAFs profile conducted by Skonieczna-Żydecka et al. [152] on a group of 116 women aged 52.0 (±4.7) years, in which 40.52% of them recognized depression, revealed a lower level of propionic acid and a higher content of isocaproic acid compared to healthy subjects. However, due to the small group size, it cannot be conclusively stated that SCAFs contribute to the depressive phenotype. Studies on animal models revealed the connection between intestinal microbiota composition and its personalities and behavior, such as anxiety or depression. Gan et al. [153] showed changes in the behavior of shy personalities of Mongolian gerbils (Meriones unguiculates) after transplantation of the gut microbiota of bold individuals. Shy gerbils often exhibited bold behavior after “bold fecal microbiota” transplantation, suggesting the association between the gut microbiota and the host’s personality.

### 5.2. Schizophrenia (SCZ)

Schizophrenia is a multifactorial disorder involving emotional, occupational, and cognitive impairments [154]. Due to cardiovascular, metabolic, and infectious diseases, adults with SCZ are at risk of early death. For individuals in the United States with SCZ, the average potential life lost is estimated to be 28.5 years [155]. According to Owen et al. [156], SCZ demonstrated three different dimensions determined as positive symptoms (hallucinations and loss of contact with reality), negative symptoms (decrease in motivation and withdrawal), and cognitive weakness (limited efficiency compared to controls).

The results of biochemical and neuroimaging studies also try to explain the etiopathogenesis of SCZ. So far, it has been possible to establish some dependencies in the systems of certain neurotransmitters, which, at least to an extent, may explain the development of clinical symptoms of SCZ. The most significant seems to be the participation of dopamine [157], although, in light of recent discoveries, dopamine plays a rather indirect role in this pathophysiology. At the same time, sources of SCZ should be found in the links between other neurotransmitters [158,159]. Kozłowska et al. [160] pointed to the association of immune/inflammatory processes and the etiology of SCZ, in which host peptides/proteins called alarmins activate signaling pathways, which lead to the development of many infection-induced or sterile inflammatory diseases. There is a growing amount of evidence that points to the significant role of the glutamatergic system. This mainly concerns the neuregulin 1 gene, a substance that activates NMDA receptors, located on the 8p12 chromosome, and the G72 and G30 genes located on chromosome 13q33, the first of which acts as an activator of amino acid oxidase (D-serine amino acid oxidase inhibitor—DAOA) [161]. Recognition of these genes supports the neurodevelopmental concept of SCZ and the role of the glutamatergic system in this process [162].

Today, it is known that both the increase in dopaminergic transmission in the mesolimbic part and the inhibition of glutamatergic transmission play an important role in the pathogenesis of positive symptoms of SCZ. Notably, the increasing number of premises proves that kynurenic acid (KYNA) may be a modulator of both these mechanisms [163]. KYNA is a nonselective antagonist of ionotropic receptors for excitatory amino acids: the NMDA receptor and the kainic acid receptor, and it is the AMPA receptor and also an antagonist of the strychnine-independent glycine site in the NMDA receptor complex. Research reveals that the level of KYNA in the CSF of patients with SCZ is elevated. Thanks to KYNA research, more and more is known about its potential role in the physiology and pathology of the CNS. However, the mechanism by which KYNA affects CNS function and, thus, the clinical picture of various diseases has not been directly described. However, significant differences in KYNA concentration in sick and healthy people indicate the participation of this compound in the pathogenesis of neurological and psychiatric diseases [164]. Because KYNA poorly penetrates the blood–brain barrier and is difficult to determine in the blood, scientists also pay attention to other metabolites of the kynurenine pathway. Recent studies have shown a 3-hydroxykynureine predictable concentration value concerning the reduction of psychopathological symptoms during the treatment of the first episode of SCZ. This offers great hope in finding biological factors that can predict the effectiveness of antipsychotic drugs. Unfortunately, despite advanced research, no effective cure for psychotic disorders, including SCZ, has yet been found, just as it has not been possible to clearly indicate the factors involved in the etiopathogenesis of this mysterious and still incurable disease.

### 5.3. Bipolar Disorder (BD)

Another serious mental illness is bipolar disorder (BD), a chronic and recurrent disease characterized by the return of hypermanic episodes or subsequent depressive episodes, with some symptoms similar to SCZ [165,166]. Worldwide, it was estimated that in 2017–2019, 46 million people suffered from BD, with New Zealanders accounting for the most significant percentage [167,168]. Although the pathophysiology of BD still requires some elucidation, changes in immune-inflammatory activity, oxidative and nitrosative stress (O&NS), and neuroregulatory tryptophan catabolites (TRYCATs) have been indicated as the etiology and course of BD [169]. With a meta-analysis prepared by Hebbrecht et al. [170], TRYCAT levels measured in cerebrospinal fluid (CSF) or serum/plasma in BD patients were lower than in healthy controls. Furthermore, the impact of the gut microbiota should be considered in the development of BD. Modifying the intestinal microbiota composition of patients with BD indicates the association between GM dysbiosis and disease progression [171]. According to studies considering intestinal microbiome diversity, an increased amount of *Coriobacteriaceae* was associated with a raised cholesterol level [172], and an increased level of *Lactobacilli* contributes to the development of obesity associated with BD [173]. The low amount of *Faecalibacterium*, an autochthonous intestinal bacterium, can also be correlated with diseases [174]. In patients diagnosed with BD, the number of *Clostridiaceae* involved in the fermentation of carbohydrates leading to the production of SCFAs was four times lower than in the control group [175,176,177].

### 5.4. Autism Spectrum Disorder (ASD)

One of the most important and dangerous neurodevelopmental diseases related to the composition of the GI microbiota is autism spectrum disorder (ASD). According to the CDC’s Autism and Developmental Disabilities Monitoring Network (ADDM) estimation, approximately 1 in 44 children in the United States has been diagnosed with ASD [178,179]. ASD is characterized by unconventional behavior, difficulties in communication and building relationships, and hypo- or hypersensitivity responses to environmental sensory signals [28]. Some studies indicated genetic factors, GI abnormalities, inflammation, or other individual and exterior factors (e.g., pre- and postnatal exposure, stress, GI microbiota, or diet), although none of them is capable of absolutely elucidating this disorder [31,180,181]. According to Azouz et al. [182], in a group of 40 autistic children aged 3 to 12 years, 82.5% of them showed gastrointestinal symptoms. Furthermore, dysbiosis related to the ratio of Firmicutes and Bacteroides and the phylum number of Firmicutes, Bacteroidetes, Fusobacteria, and Verrucomicrobia was demonstrated in patients diagnosed with ASD [180]. In the same study, the authors revealed that in patients with ASD, the changes also affect the level of SCFAs and volatile organic compounds (VOC), including, among others, indole, which is a metabolite of tryptophan and the precursor of serotonin and melatonin. However, these data should be carefully interpreted due to the possible influence of antibiotic treatment or personalized diet in patients with ASD [29]. To clearly define the role of the gut microbiota in patients, further research and an entire view to connect dependences between GM and ASD are needed.

### 5.5. Attention-Deficit Hyperactivity Disorder (ADHD)

A common neurodevelopmental disorder is attention deficit hyperactivity disorder (ADHD), which affects 6 million children aged 3–17 years [183]. It manifests in difficulties maintaining attention, and sudden, unexpected behavior [184]. The genes for the dopamine receptors DRD4 and DRD5 and dopamine and serotonin transmitters are considered the main etiological factors of this disease [185]. A growing amount of evidence indicates the connection with the microbiome–gut–brain axis [186,187]. In the microbiome study conducted by Aarts et al. [188], 96 participants participated, of whom 19 had been diagnosed with ADHD and 77 were healthy. Results revealed differences in taxa, where in ADHD cases, the phylum Actinobacteria was more abundant (for example, *Bifidobacterium*), while the abundance of Firmicutes decreased. Interestingly, the same study revealed that the microbiome of cases of ADHD shows a more remarkable ability to produce cyclohexadienyl dehydratase (CDT), which is involved in synthesizing the dopamine precursor (phenylalanine).

On the contrary, the meta-analysis prepared by Wang et al. [189], which evaluated the intestinal microbiota and ADHD, did not show significant differences at the phylum and family levels beyond the higher level of *Blautia* in patients with ADHD compared to healthy control. This microorganism plays a regulatory role in metabolic and inflammatory diseases, as well as biotransformation [190]. Future research that specifies the connection between the microbiome–gut–brain axis and ADHD should cover a larger demographically diverse study group [191]. The risk of developing neuropsychiatric disorders could be reduced through probiotic supplementation in early life. Pärtty et al. [192] showed that early administration of *Lactobacillus rhamnosus* GG might decrease the risk of ADHD. It was shown that *L. rhamonsus*, through the vagus nerve, regulated emotional behavior and the central GABA receptor expression in a mouse [193]. In addition, the influence of dietary patterns on ADHD patients is important. Since food contains artificial color additives, to decrease the hyperactive behavior of ADHD patients, it is necessary to exclude such products from the diet [194]. Intake of omega-3 PUFAs is also significant, in particular, docosahexaenoic acid (DHA) and eicosapentaenoic acid (EPA), as both are essential for proper membrane fluidity, neurotransmission, and receptor function [195]. In a study based on animal male models of ADHD, nutrition individuals with a diet enriched in omega-3 PUFAs caused decreased impulsivity and improved concentration [196].

## 6. Future Perspectives

In summary, the experimental data described in this review confirm that disturbances in the composition of the microbiota lead to the development of mental and psychiatric disorders in the host organism. Knowledge about different communication pathways on the bidirectional gut–brain axis significantly impacts the development of new therapeutic strategies because the gut microbiota undergoes modifications even in utero. Additionally, the dietary composition modulates GM and, thus, the availability of their metabolites in the intestine. By analyzing studies on probiotic supplementation, we can observe the relationships between the microbiota and mood disorders and the disease-alleviating effect. This, in turn, enables the design of alternative therapeutic strategies for the treatment of mood disorders. Current scientific reports are based mainly on animal models. Numerous studies are also required on people with mood disorders, showing probiotics’ effect.

## 7. Conclusions

Our review of the current literature presents bidirectional communication between the gut and the brain through the microbiome–gut–brain axis. In this work, we focus on the pathogenesis of depression, schizophrenia, and bipolar disorder and some factors affecting the formation of gut microbiota. The COVID-19 pandemic has led to an increase in the number of people suffering from mental disorders. Considering the fact that the gut microbiota is altered in people with various mental disorders and bearing in mind that intestinal dysbiosis is associated with the development of inflammation, which is related to the forming and progression of symptoms of these diseases, there is a need to expand knowledge about bacterial species in larger groups of people with mental disorders, including the effects of medications and eating habits. Hence, expanding the knowledge on the association between gut–microbiota–brain, and pathways involved in this communication, is essential to develop protective strategies or create new therapeutic approaches against mental disorders.

## Data Availability

Not applicable.
